# Surveillance of vancomycin-resistant enterococci reveals shift in dominating clones and national spread of a vancomycin-variable *vanA Enterococcus faecium* ST1421-CT1134 clone, Denmark, 2015 to March 2019

**DOI:** 10.2807/1560-7917.ES.2019.24.34.1900503

**Published:** 2019-08-22

**Authors:** Anette M Hammerum, Ulrik S Justesen, Mette Pinholt, Louise Roer, Hülya Kaya, Peder Worning, Sanne Nygaard, Michael Kemp, Marianne Engell Clausen, Karen Leth Nielsen, Jurgita Samulioniené, Mona Kjærsgaard, Claus Østergaard, John Coia, Turid Snekloth Søndergaard, Shahin Gaini, Kristian Schønning, Henrik Westh, Henrik Hasman, Barbara Juliane Holzknecht

**Affiliations:** 1 Department for Bacteria, Parasites and Fungi, Statens Serum Institut, Copenhagen, Denmark; 2 Department of Clinical Microbiology, Odense University Hospital, Odense, Denmark; 3 Department of Clinical Microbiology, Hvidovre University Hospital, Hvidovre, Denmark; 4 Department of Clinical Microbiology, Herlev and Gentofte University Hospital, Herlev, Denmark; 5 Department of Clinical Microbiology, Slagelse Hospital, Slagelse, Denmark; 6 Department of Clinical Microbiology, Rigshospitalet, Copenhagen, Denmark; 7 Department of Clinical Microbiology, Aalborg University Hospital, Aalborg, Denmark; 8 Department of Clinical Microbiology, Aarhus University Hospital, Aarhus, Denmark; 9 Department of Clinical Microbiology, Lillebaelt Hospital, Vejle, Denmark; 10 Department of Clinical Microbiology, Hospital South West Jutland, Esbjerg, Denmark; 11 Department of Clinical Microbiology, Hospital Sønderjylland, Sønderborg, Denmark; 12 Medical Department, National Hospital Faroe Islands, Torshavn, Faroe Islands; 13 Department of Infectious Diseases, Odense University Hospital, Odense, Denmark; 14 Centre of Health Research, University of the Faroe Islands, Torshavn, Faroe Islands; 15 Institute of Clinical Medicine, University of Copenhagen, Copenhagen, Denmark

**Keywords:** VRE, VVE, Enterococci, vanA, vanB, MLST

## Abstract

We describe clonal shifts in *vanA Enterococcus faecium* isolates from clinical samples obtained from patients in Denmark from 2015 to the first quarter (Q1) of 2019. During Q1 2019, the vancomycin-variable enterococci (VVE) ST1421-CT1134 *vanA E. faecium* became the most dominant *vanA E. faecium* clone and has spread to all five regions in Denmark. Among 174 *E. faecium* isolates with *vanA, vanB* or vanA/*vanB* genes in Q1 2019, 44% belonged to this type.

We describe the clonal shift for *vanA Enterococcus faecium* during the last 4 years and the national spread of a vancomycin-variable *vanA E. faecium* ST1421-CT1134 clone in Denmark. The aim is to highlight the importance of using molecular methods for detecting vancomycin-variable enterococci (VVE), and to alert other countries about this emerging nosocomial clone.

## Vancomycin-variable enterococci

Vancomycin-variable enterococci (VVE) are *E. faecium* harboring the *vanA* gene complex, but being phenotypically vancomycin susceptible [1,2]. VVE can only be detected by molecular methods and cannot be cultured on selective vancomycin-containing media. Different clones of VVE have caused nosocomial outbreaks and development of vancomycin-resistant revertant mutants *in vitro* and *in vivo* has been described [1,3-5]. This makes the detection of VVE highly important in clinical samples in order to assure relevant antibiotic treatment and in screening samples to avoid nosocomial spread. In 2015 and 2016, sporadic VVE with different genetic background were detected in the Capital Region of Denmark, in connection with vancomycin-resistant enterococci (VRE) outbreaks (data not shown). In 2016, a VVE clone belonging to ST1421-CT1134, which displays variable vancomycin susceptibility (minimum inhibitory concentration (MIC) 1 to ≥ 256 mg/ml) was detected in screening samples from a hospital in the Capital Region [5]. One strain, Efm-V1511, belonging to this clone was characterised by Hansen et al. [5]. Efm-V1511 had a 49.6 Kp plasmid, which carried the Tn*1546* (*vanA* transposon). Tn*1546* was truncated in *vanX* by a 252 bp 3' deletion explaining the vancomycin susceptibility of Efm-V1511. In ST1421-CT1134 isolates resistant to vancomycin, resistance could be attributed to changes in *ddl* disrupting gene function sometimes accompanied by changes in *vanS*, increased pHVH-V1511 copy number or the existence of an additional *vanA*-containing plasmid encoding a functional *vanX* [5].

## National surveillance of vancomycin-resistant and vancomycin-variable enterococci

**Figure 1:**
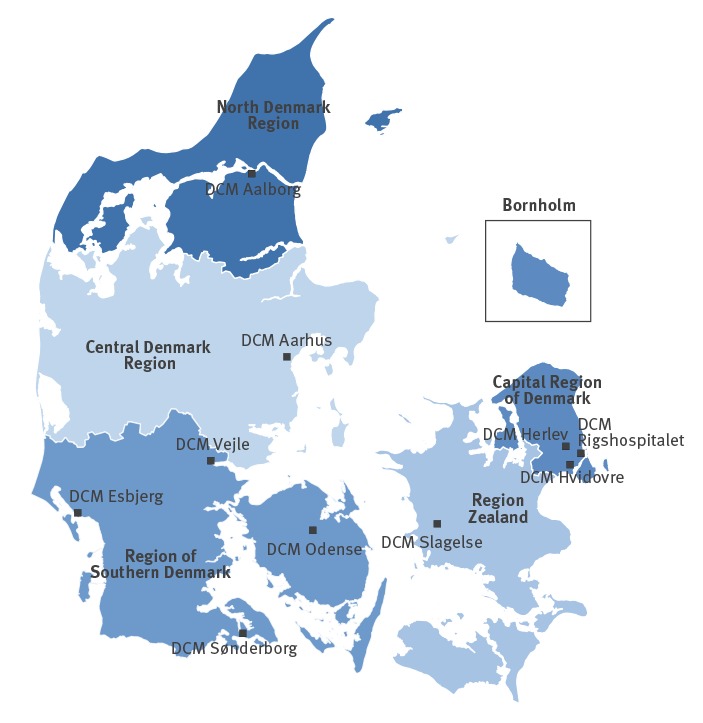
The five healthcare regions and the 10 Departments of Clinical Microbiology, Denmark, 2019

DCM: Department of Clinical Microbiology; ND: not detected.

Modified from DANMAP 2017 [7].

We have previously described the surveillance of vancomycin-resistant enterococci (VRE) in clinical isolates in Denmark from 2005 to 2015 [6]. In the present study, we follow up and describe the data from isolates obtained from 2016 through the first quarter (Q1) of 2019. Since 2005, VRE isolates from clinical samples, e.g. urine, blood and tissue, as opposed to screening (faecal) isolates have been voluntarily submitted to Statens Serum Institut (SSI) from Danish Departments of Clinical Microbiology (DCM) for species identification, genotyping and surveillance (Figure 1) [7]. Only one isolate per patient per 12 months was included. All VRE isolates (699 *E. faecium* and 30 *E. faecalis*) were tested for the presence of vancomycin resistance genes *vanA* and *vanB* by PCR from 2005 through 2014.

From 2015 through Q1 2019, all clinical VRE/VVE isolates (n = 1,935) underwent whole-genome sequencing (WGS) as previously described [6]. From the WGS data, multilocus sequence type (MLST), and *van* genes were extracted *in silico*. The isolates were further subtyped in SeqSphere + (Ridom GmbH, Münster, Germany (http://www.ridom.de/seqsphere/)) using the cgMLST scheme by de Been et al. [8] for *E. faecium*.

VVE diagnostic algorithms have differed substantially over time and between the five Danish regions. In 2017, testing of phenotypically vancomycin-susceptible *E. faecium* isolates from blood cultures for the presence of *vanA*/*vanB* genes by PCR was introduced in the DCMs in the Capital Region. During 2018, this was expanded to testing of all clinical *E. faecium* isolates. During 2018, molecular testing by PCR of *E. faecium* from all clinical samples was also implemented in one of the four DCMs in the Region of Southern Denmark. Furthermore, *E. faecium* isolates from blood cultures were tested by PCR for *vanA/vanB* genes in another DCM in the Region of Southern Denmark and in the DCM in the Central Denmark Region in 2018. In Q1 2019, diagnostic algorithms to detect VVE have expanded. Most of the DCMs across Denmark test at least all blood culture *E. faecium* isolates for the presence of *vanA* genes using PCR.

## *Enterococcus faecium* and *Enterococcus faecalis* isolates from clinical samples carrying *vanA* and *vanB* genes

**Figure 2:**
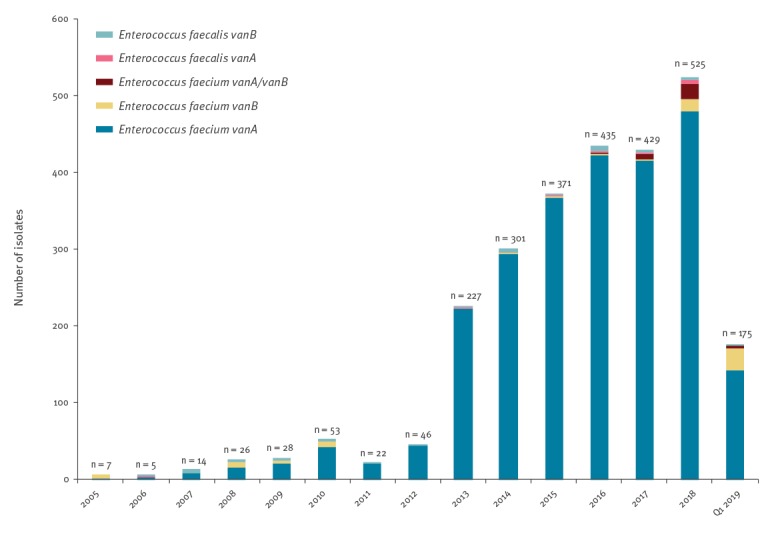
Vancomycin-resistant and vancomycin-variable *Enterococcus faecium* and vancomycin-resistant *E. faecalis* isolates from clinical samples carrying *van* genes, Denmark, 2005–Q1 2019 (n = 2,664)

From 2005 to Q1 2019, 2,503 *vanA E. faecium*, 74 *vanB E. faecium*, 32 *vanA/vanB E. faecium*, 12 *vanA E. faecalis,* and 43 *vanB E. faecalis* from clinical samples were submitted to SSI (Figure 2).

Q1: first quarter.

## Emergence and disappearance of major *Enterococcus faecium* clones

Of the 1,935 VRE/VVE isolates obtained from 2015 through Q1 2019, 1,910 were *E. faecium* and 25 *E. faecalis* (Figure 2).

The *E. faecium* isolates belonged to 29 sequence types (STs). ST80 (22%), ST203 (65%) and ST1421 (9%) were most prevalent. Typing by cgMLST revealed 156 different complex types (CTs).

The 13 most common types of *vanA,* *vanB* and *vanA/vanB* *E. faecium* from 2015 to Q1 2019 are shown in Table 1. From 2015 to 2019, three types were dominating: ST80-CT14 *vanA E. faecium*, ST203-CT859 *vanA E. faecium* and ST1421-CT1134 *vanA E. faecium* (Table 1).

**Table 1:** Description of the most common types of *vanA* and/or *vanB* *Enterococcus faecium* by MLST and cgMLST, Denmark, 2015–Q1 2019 (n = 1,910)

Types	2015 (n = 369)		2016 (n = 427)		2017 (n = 425)		2018 (n = 515)		Q1 2019 (n = 174)	
	n	%	n	%	n	%	n	%	n	%
ST80-CT14 *vanA*	81	22	38	9	15	4	1	< 1	ND	ND
ST80-CT24 *vanA*	23	6	19	4	11	3	2	< 1	4	2
ST80-CT860 *vanA*	7	2	11	3	ND	ND	ND	ND	ND	ND
ST80-CT866 *vanA*	14	4	10	2	7	2	ND	ND	ND	ND
ST80-CT991 *vanA*	ND	ND	11	3	9	2	6	1	ND	ND
ST80-CT1160 *vanA*	ND	ND	ND	ND	7	2	10	2	ND	ND
ST80-CT1064 *vanA/vanB*	ND	ND	2	< 1	8	2	23	5	4	2
ST80-CT1729 *vanA*	ND	ND	ND	ND	ND	ND	22	4	2	1
ST117-CT873 *vanA*	5	1	12	3	ND	ND	ND	ND	ND	ND
ST117-CT1180 *vanA*	ND	ND	ND	ND	9	2	30	6	7	4
ST117-CT36 *vanB*	ND	ND	ND	ND	ND	ND	2	< 1	16	9
ST203-CT859 (subtypes CT1051 and CT1507) *vanA*	188	51	271	64	265	63	161	31	20	12
ST1421-CT1134 *vanA*	ND	ND	2	< 1	13	3	176	34	77	44
Other types	51	14	51	12	81	19	82	16	44	25

CT: cluster type (cgMLST); MLST: multilocus sequence typing; ND: not detected; ST: sequence type (MLST); Q1: first quarter.

In 2015, 22% of the *E. faecium* isolates belonged to ST80-CT14 *vanA E. faecium.* The type decreased during 2016.

ST203-CT859 *vanA E. faecium* isolates were first detected during the end of 2014 [6]. It emerged very fast and was the most prevalent *vanA E. faecium* type (together with its subtypes CT1051 and CT1507) during 2015 to 2017, but decreased in 2018 (Table1). In Q1 2019 only 12% of the VRE/VVE *E. faecium* isolates belonged to ST203-CT859.

In 2017, 3% of the *E. faecium* isolates belonged to the VVE clone, ST1421-CT1134 *vanA E. faecium.* This type was only detected from clinical samples from the Capital Region. In 2018, 34% of the *E. faecium* isolates belonged to ST1421-CT1134 and were detected in the Capital Region, the Region Zealand and from one DCM in the Region of Southern Denmark (Table 1, Table 2). During Q1 2019, ST1421-CT1134 *vanA E. faecium* was the most prevalent type (44%) (Table 1). It was detected in all five regions of Denmark, 50 isolates from the Capital Region, one isolate from Region Zealand, 23 isolates from the Region of Southern Denmark, two isolates from Central Denmark Region and one isolate from the North Denmark Region (Table 2). Furthermore, ST1421-CT1134 *vanA E. faecium* spread to the Faroe Islands during 2018 and 2019 (data not shown).

**Table 2:** Regional occurrence of ST1421-CT1134 *vanA E. faecium,* Denmark, 2016–Q1 2019 (n = 268)

Region	2016 (n = 2)	2017 (n = 13)	2018 (n = 176)	Q1 2019 (n = 77)
Capital Region of Denmark	2	9	158	50
Region Zealand	ND	3	9	1
Region of Southern Denmark	ND	ND	9	23
Central Denmark Region	ND	ND	ND	2
North Denmark Region	ND	1	ND	1

Q1: first quarter.

## Discussion and conclusion

During 2005 to Q1 2019, most of the Danish clinical VRE isolates have been *vanA E. faecium* isolates. This study shows that predominating clones shifted over time and, importantly, the emergence of a vancomycin-variable clone, ST1421-CT1134 *vanA E. faecium*, that has spread to all the five Danish regions in 2019.

Although the *E. faecium* isolates belonged to 156 CTs, three types (ST80-CT14 *vanA E. faecium,* ST203-CT859 *vanA E. faecium,* ST1421-CT1134 *vanA E. faecium*) have dominated during the last 4 years.

ST80-CT14 *vanA E. faecium* was highly prevalent in the Capital Region during 2012 to 2015 [9]. The *vanA E. faecium* constituting Group2_ST80 in the paper by Pinholt et al. [9] belonged to ST80-CT14 (data not shown). On a national level, the numbers of ST80-CT14 *vanA E. faecium* decreased during 2016 to 2018, and this clone was not detected during Q1 2019.

ST203-CT859 *vanA E. faecium* emerged during 2015 through 2017 and nearly disappeared 2019. This clone has spread to Sweden, the Faroe Islands and Greenland [6,7].

Because of differences in diagnostic algorithms, there is a detection bias of VVE. It seems very likely that ST1421-CT1134 *vanA E. faecium* have been under-reported in some regions at least during some periods. Thus, the rising incidence could partly be explained by increasing molecular testing of vancomycin susceptible isolates. However, a sharply increasing incidence has also been seen in DCM with extensive testing for VVE.

The origin of ST80-CT14 *vanA E. faecium* and ST203-CT859 *vanA E. faecium* are still unknown. *vanA E. faecium* isolates belonging to ST1421-CT1134 have also been reported from Australia, but these isolates have not been VVE [10]. Why these three clones were so successful is unknown.

The spread of the VVE clone, ST1421-CT1134 *vanA E. faecium*, in Denmark is of concern, especially since VVE diagnostic is challenging. Because of this, the clone is likely to be underdiagnosed, which facilitates further spread. Since cross-border spread has been described for VRE, countries with patients transferred from Denmark should be aware of the vancomycin-variable ST1421-CT1134 *vanA E. faecium* clone.

## References

[1] Sivertsen A Pedersen T Larssen KW Bergh K Rønning TG Radtke A A Silenced vanA Gene Cluster on a Transferable Plasmid Caused an Outbreak of Vancomycin-Variable Enterococci. Antimicrob Agents Chemother. 2016;60(7):4119-27. 10.1128/AAC.00286-16 27139479 PMC4914660

[2] Kohler P Eshaghi A Kim HC Plevneshi A Green K Willey BM Toronto Invasive Bacterial Diseases Network (TIBDN) Prevalence of vancomycin-variable Enterococcus faecium (VVE) among vanA-positive sterile site isolates and patient factors associated with VVE bacteremia. PLoS One. 2018;13(3):e0193926. 10.1371/journal.pone.0193926 29566004 PMC5863957

[3] Szakacs TA Kalan L McConnell MJ Eshaghi A Shahinas D McGeer A Outbreak of vancomycin-susceptible Enterococcus faecium containing the wild-type vanA gene. J Clin Microbiol. 2014;52(5):1682-6. 10.1128/JCM.03563-13 24523464 PMC3993680

[4] Coburn B Low DE Patel SN Poutanen SM Shahinas D Eshaghi A Vancomycin-variable Enterococcus faecium: in vivo emergence of vancomycin resistance in a vancomycin-susceptible isolate. J Clin Microbiol. 2014;52(5):1766-7. 10.1128/JCM.03579-13 24523476 PMC3993649

[5] Hansen TA Pedersen MS Nielsen LG Ma CMG Søes LM Worning P Emergence of a vancomycin-variable Enterococcus faecium ST1421 strain containing a deletion in vanX. J Antimicrob Chemother. 2018;73(11):2936-40. 10.1093/jac/dky308 30113682

[6] Hammerum AM Baig S Kamel Y Roer L Pinholt M Gumpert H Emergence of vanA Enterococcus faecium in Denmark, 2005-15. J Antimicrob Chemother. 2017;72(8):2184-90. 10.1093/jac/dkx138 28541565

[7] The Danish Integrated Antimicrobial Resistance Monitoring and Research Programme (DANMAP). DANMAP 2017 - Use of antimicrobial agents and occurrence of antimicrobial resistance in bacteria from food animals, food and humans in Denmark. Copenhagen: DANMAP; 2017. Available from: https://www.danmap.org/-/media/arkiv/projekt-sites/danmap/danmap-reports/danmap-2017/danmap2017.pdf?la=en.

[8] de Been M Pinholt M Top J Bletz S Mellmann A van Schaik W Core Genome Multilocus Sequence Typing Scheme for High- Resolution Typing of Enterococcus faecium. J Clin Microbiol. 2015;53(12):3788-97. 10.1128/JCM.01946-15 26400782 PMC4652124

[9] Pinholt M Bayliss SC Gumpert H Worning P Jensen VVS Pedersen M WGS of 1058 Enterococcus faecium from Copenhagen, Denmark, reveals rapid clonal expansion of vancomycin-resistant clone ST80 combined with widespread dissemination of a vanA-containing plasmid and acquisition of a heterogeneous accessory genome. J Antimicrob Chemother. 2019;74(7):1776-85. 10.1093/jac/dkz118 30929020

[10] van Hal SJ Beukers AG Timms VJ Ellem JA Taylor P Maley MW Relentless spread and adaptation of non-typeable vanA vancomycin-resistant Enterococcus faecium: a genome-wide investigation. J Antimicrob Chemother. 2018;73(6):1487-91. 10.1093/jac/dky074 29566173

